# Exposure to Secondhand Tobacco Smoke and Interventions Among Pregnant Women in China: A Systematic Review

**DOI:** 10.5888/pcd12.140377

**Published:** 2015-03-19

**Authors:** Liying Zhang, Jason Hsia, Xiaoming Tu, Yang Xia, Lihong Zhang, Zhenqiang Bi, Hongyan Liu, Xiaoming Li, Bonita Stanton

**Affiliations:** Xiaoming Li, Bonita Stanton, Pediatrics Prevention Research Center, Carman and Ann Adams Department of Pediatrics, Wayne State University School of Medicine, Detroit, Michigan; Xiaoming Tu, School of Public Health, Nanjing Medical University, Nanjing, China; Jason Hsia, Yang Xia, Centers for Disease Control and Prevention, Atlanta, Georgia; Lihong Zhang, Shandong Provincial Key Laboratory for Reproductive Genetics and Reproductive Technologies, Jinan, China; Zhenqiang Bi, Shandong Provincial Center for Disease Control and Prevention, Jinan, China; Hongyan Liu, National Health and Family Planning Commission of China, Beijing, China.

## Abstract

**Introduction:**

Smoking prevalence is high among men in China. One result is that a large number of nonsmoking Chinese women may be exposed daily to secondhand smoke (SHS). Exposure is particularly problematic for pregnant women because of potential adverse reproductive effects. To determine the extent of this exposure and to summarize existing intervention studies designed to reduce SHS exposure in China, a systematic review of the literature published from 1995 through 2012 was conducted.

**Methods:**

We searched the PubMed and Wanfang databases for studies published from 1995 through 2012 using various search terms including SHS, pregnant women, and China. Only articles on prevalence of SHS exposure and interventions to reduce exposure to SHS were selected.

**Results:**

We identified 132 studies during the initial searches. Eight of 13 eligible studies reported the prevalence of SHS exposure among pregnant women; estimates ranged from 38.9% to 75.1%. Few SHS prevention interventions among pregnant women in China have been studied; we found only 5 such studies. The interventions primarily focused on changing husbands’ smoking behaviors; some interventions focused on women’s avoidance behaviors.

**Conclusion:**

Prevalence of exposure to SHS among pregnant women is high in China. Information is limited on effective interventions to protect pregnant women from exposure. The results of this review can provide the basis for the design and evaluation of interventions to help pregnant women avoid SHS exposure.

## Introduction

Globally, about 35% of female nonsmokers are involuntary smokers and are exposed to secondhand tobacco smoke (1). Secondhand smoke (SHS) has been identified as a human carcinogen by the International Agency for Research on Cancer (2). The number of deaths due to exposure to SHS is estimated to be approximately 600,000 each year worldwide (3). SHS exposure can cause cardiovascular disease, lung cancer, numerous health problems in infants and children, and adverse reproductive outcomes (4). For example, maternal exposure to SHS during pregnancy decreases infant birth weight and is associated with increased risk of preterm delivery (5).

China produces and consumes more tobacco than any other country in the world (6). Among men aged 15 to 69, national estimates of the prevalence of current smoking are high (63.0% in 1996, 57.4% in 2002, and 54.0% in 2010), whereas the estimates among women are low (3.8% in 1996, 2.6% in 2002, and 2.0% in 2010) (7–9). Although prevalence estimates come from surveys using different sample designs and operation protocols, these patterns have not changed substantially in more recent years. The 2010 Global Adult Tobacco Survey in China reported that 65.1% of nonsmoking women of childbearing age (15–49 y) were exposed to SHS at home and 52.6% were exposed in the workplace (10). According to the National Bureau of Statistics of China, there were 380 million women aged 15 to 49 and an estimated 100 million pregnant women in China in 2010 (11). Therefore, the public health implications of SHS exposure during pregnancy are substantial.

China ratified the World Health Organization’s Framework Convention on Tobacco Control (FCTC), but it faces the challenge that smoking is the social norm. Although passing smoke-free legislation, promoting pictorial warning labels, and raising sales taxes for tobacco products are all important components of tobacco control in China, changing the social norm of tobacco use is also important. Some potential breakthrough points in changing the social norm are the establishment of smoke-free hospitals and the promotion of health professionals as models for health behaviors. Joint-venture business offices could implement programs in which smoking employees sacrifice higher-paying jobs for smoking in the office. Preventing pregnant women from exposure to SHS is another potential breakthrough point because pregnancy is a special time for health. The public health implications of SHS exposure during pregnancy for women and their babies are substantial. The objective of this review was to summarize the scientific literature from 1995 through 2012 on the prevalence of exposure to SHS among pregnant women in China and on intervention studies designed to reduce SHS exposure among this population.

## Methods

Several methods were used to find and select relevant publications. Studies were selected and retrieved by searching articles published between January 1, 1995, and December 31, 2012, and indexed by PubMed with a combination of the key words “secondhand smoke,” “passive smoking,” “involuntary smoking,” or “environmental tobacco smoke” and the key words “pregnant women” and “China.” Studies were also selected and retrieved by searching in Wanfang Data, a database of Chinese literature covering 8,000 core scientific journals in Chinese. Additionally, we searched with a combination of the key words used in PubMed (PubMed Advanced Search Builder). The criteria for inclusion were articles that described either prevalence of SHS exposure among pregnant women or results of an intervention to reduce exposure to SHS among pregnant women. Additionally, prevalence studies were included only when the margin of error for key SHS exposure variables was 0.05 or less. Any articles that did not meet these criteria were excluded. The search results were reviewed, and articles in English and Chinese were selected on the basis of the abstracts and titles; those that appeared to be relevant were reviewed further. We also reviewed all relevant articles cited in the reference lists of the 12 articles initially selected for review (Figure). Dissertations, presentation abstracts, letters to the editor, editorials, news articles, and unpublished reports were not reviewed. We initially identified 132 study abstracts (Figure). Two trained individuals independently abstracted relevant information onto coding sheets using a standard protocol and met to review their findings and reach consensus. 

**Figure:**
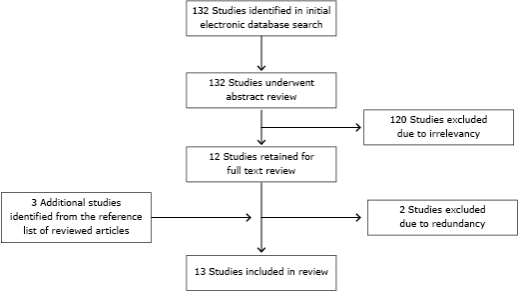
Selection of studies in review of articles on exposure to secondhand smoke among pregnant women in China, 1995–2012.

## Results

### Study characteristics

We found 13 studies: 8 studies reported the prevalence of SHS exposure among pregnant women, and 5 studies reported on interventions (Table 1 [12–19] and Table 2 [20–24]). Three of the prevalence studies were population-based (13–15), 4 studies were hospital-based (16–19), and 1 study was community-based (conducted in Shanghai) (12). All studies had similar cross-sectional study designs: they defined a study region, such as a province or a city, within which hospitals and maternal and child medical centers were chosen either randomly (population-based studies) or according to criteria representative or feasible (hospital-based studies). From those hospitals and medical centers, pregnant women were randomly selected. The studies were conducted in Beijing, Changchun, Guangzhou, Changsha, Macao, or Sichuan province, and the sample sizes ranged from 200 to 2,770, with median of 1,066 participants. All pregnant women who came to the study hospitals for their first prenatal visit during the study period were invited to participate. In the community-based study, women who brought their infants for vaccination to any of 3 community health centers in Shanghai between June 2005 and August 2006 were invited to participate in the study. A total of 950 women were enrolled, and face-to-face interviews were used to collect information on SHS exposure.

**Table 1:** Summary of Studies on Prevalence of Exposure to Secondhand Smoke (SHS) Among Pregnant Women in China

Authors, Year of Publication	Study Site (Study Years)	Study Design	Study Setting and Sample Size	Measures	Prevalence of SHS Exposure	Women’s Avoidance Behaviors
Fu et al (12), 2008	Shanghai (2005–2006)	A cross-sectional study; a population-based survey	New mothers with infants aged 5–8 months in 3 communities (N = 950); 701 nonsmokers included in the analysis; face-to-face interviews; self-administered questionnaires	Frequency, amounts, and time of exposure before, during, and after pregnancy	41.9% in total; 73.0% at home; 65.0% in workplace; 83.3% among others (friends, relatives, customers, strangers [in public places])	31.4% always tried to stop their husbands from smoking in their presence; 31.0% tried to stop family members from smoking; 23.5% tried to stop others from smoking
Di et al (13), 2010	Henan (no study year reported)	A cross-sectional study; a population-based survey	Nonsmoking pregnant women in 6 hospitals (N = 1,660)	Prevalence of exposure and related factors	68.3% overall; 66.2% at home; 36.1% in workplace; 56.3% in public places	None reported
Yang et al (14), 2010	Chengdu (2008)	A cross-sectional study; a population-based survey	Nonsmoking pregnant women in 8 hospitals (N = 1,181); 784 resided in rural areas and 397 resided in urban areas	Sources of exposure, duration of exposure; hair nicotine test	75.1% in total; 57.7% at home; 11.6% in workplace; 21.4% in public places	None reported
Luo et al (15), 2011	Macao (2009)	A cross-sectional study; a population-based survey	Nonsmoking pregnant women in a hospital (N = 200)	Knowledge and attitudes; avoidance actions	17.0% at home; 59.3% in workplace; 28.0% in public places	69.4% stopped husbands from smoking in their presence; 47.8% stopped family members from smoking in their presence; 18.3% stayed away from husbands; 36.8% stayed away other family members; 10.0% opened windows when husbands smoked in their presence; 11.7% opened windows when other family members smoked in their presence
Loke et al (16), 2000	Guangzhou (1996–1997)	A cross-sectional study; a hospital-based survey	Nonsmoking pregnant women in a hospital (N = 1,449); 872 had husbands who smoked and 577 had husbands who did not smoke	Knowledge and attitudes toward SHS; hours of exposure per day at home, in public places, at work; whether nonsmoking women moved away from smoker, asked smoker to move, or asked smoker to stop smoking	71.0% at home; 60.0% in the workplace; 77.0% in public places	At home, 52.2% moved from smoker, 25.7% asked smoker to move. In public places, 74.5% often moved from smoker. In a restaurant, 51.4% thought to change to another restaurant.
Sun et al (17), 2010	Changsha (2005)	A cross-sectional study; a hospital-based survey in 5 preselected hospitals	Nonsmoking pregnant women in 5 hospitals (N = 620)	Prevalence of exposure and related factors	38.9% overall; 36.9% at home; 19.1% in workplace; 17.8% in public places	Persuaded smoker to quit at home (38.4%); persuaded smoker to quit in workplace (20.3%); 70.2% stayed away from smoker at home; 25.9% stayed away from smoker in workplace
Zhou et al (18), 2011	Changsha (2010)	A cross-sectional study; a hospital-based survey in 4 preselected hospitals	Nonsmoking pregnant women in 4 hospitals (N = 641)	Exposure per day; knowledge and attitudes toward SHS; self-efficacy of stopping others from smoking	61.7% overall	None reported
Lee et al (19), 2012	Beijing and Changchun (2000–2001)	A cross-sectional study; a hospital-based survey in 4 preselected hospitals (2 from the northern and 2 from the southern part of China)	New mothers in 4 major hospitals (N = 2,770); 1,363 in Beijing and 1,407 in Changchun	Average number of hours of exposure per day; number of days of exposure per week; number of cigarettes per day husband smoked; pregnancy complications and neonatal outcomes	53.0% overall; 22.8% at home; 31.7% in workplace; 11.0% in public places	None reported

**Table 2:** Summary of Studies of Interventions on Exposure to Secondhand Smoke (SHS) Among Pregnant Women in China

Authors, Year of Publication	Study Site (Study Years)	Intervention	Study Setting and Sample Size	Type of Intervention	Measures	Intervention Outcomes
Gao et al (20), 2004	Guangzhou (1996–1997)	A randomized controlled trial	Nonsmoking pregnant women with smoking husbands in a hospital (N = 1,532); intervention group (n = 380) and control group (n = 378)	Education: intervention group received education from doctors on avoidance behaviors and how to persuade smoking husband to quit; brochures provided	Frequency of women moving from smoker; frequency of persuading husbands to quit; frequency of husbands attempting to quit; changes in the number of cigarettes smoked by husbands	Higher percentage of avoidance behavior (women walked away from smokers) in intervention group than in control group (44.5% vs 39.7%, *P* = .05). The proportion of pregnant women stopping husbands from smoking was higher in intervention group (71.6% vs 55.6%, *P *< .01). The proportion of husbands attempting to quit was higher in intervention group (45.5% vs 32.8%, *P* < .01). The proportion of husbands who quit smoking for at least 7 days was higher in intervention group than in control group (8.7% vs 4.5%, *P* < .05).
Loke and Lam (21), 2005	Guangzhou (1996–1997)	A randomized controlled trial; used a Reasoned Action Model	Nonsmoking pregnant woman with smoking husbands in a hospital (N = 758); intervention group (n = 380) and control group (n = 378); follow-up took place within 1 month of expected delivery date.	Education: intervention group received standardized advice from doctors on recognizing health risks of exposure, avoiding exposure, and helping husbands to quit	Husbands’ attempts to give up smoking in the past 7 days; husbands’ change in the number of cigarettes smoked per day; husbands’ giving up totally for 1 month or longer; Husbands’ attempts at giving up and actually giving up.	More husbands in the intervention group than in the control group (30.0% vs 22.2%, *P* < .01) attempted to give up; more husbands in the intervention group than in the control group (39.7% vs 17.7%, *P* < .01) reduced their amount of smoking; more husbands quit smoking for 1 month in the intervention group than in the control group (6.1% vs 4.2%, *P* = .26)
Yang and Mao (22), 2010	Sichuan (2008)	A randomized controlled trial	Nonsmoking pregnant women in 8 hospitals (N = 186); intervention group (n = 91) and control group (n = 95). Used a biomarker (hair ) to measure level of exposure to SHS	Education: provided brochures, lecture, role play, video, hotline, knowledge competition	Knowledge and skills of avoidance; the number of cigarettes smoked per day by husbands; hair nicotine test	The number of cigarettes per day that husbands smoked in intervention group decreased significantly compared with the control group (*P* < .01); proportion of families creating smoke-free restriction policy increased in intervention group (*P* < .01); hair nicotine concentration decreased by 0.2 (log µɡ/ɡ) in intervention group and increased by 0.1(log µɡ/ɡ) in control group (*P *< .01)
Lee (23), 2008	Chengdu (2007)	Pretest–posttest intervention; the Health Belief Model	Nonsmoking pregnant women in 3 hospitals for prenatal care whose husbands were smokers; Pretest–posttest group discussions (n = 55); pretest–posttest intervention (n = 128); follow-up at 16 weeks	Education: authoritative figures from the hospital gave motivational speeches. Video used to communicate knowledge; role play used to instill a feeling of efficacy; booklet used to communicate knowledge and teach skills; telephone hotline used for counseling and reinforcement	Changes in knowledge, attitudes, and avoidance actions	A higher proportion of women asked smokers to stop smoking in their presence (98.4% vs 92.2%, *P* < .05 for husband; 86.7% vs 56.2%, *P* < .01 for other family members) at postintervention compared with preintervention
Hu et al (24), 2011	Changsha (2011)	A pretest-posttest intervention	Nonsmoking pregnant women in 4 hospitals (N = 1,015); retention rate was 77.7%	Education: mass media (radio, newspaper); video; role play	Knowledge and skills of avoidance	Prevalence of SHS exposure declined from 62.2% in preintervention to 33.8% at postintervention (*P* < .01); self-efficacy of stopping others smoking increased at postintervention (*P* < .01)

Of the 5 intervention studies (Table 2), 3 were randomized controlled trials (RCTs) (20–22) and 2 were pretest–posttest studies (23,24). The sample sizes for the 3 RCT intervention studies were 1,532, 758, and 186, and the sample sizes for the 2 pretest–posttest studies were 128 and 1,015. One was guided by Fishbein-Ajzen’s Reasoned Action Model (21) and one by the Health Belief Model (23). The other 3 studies did not mention a theoretical basis (20,22,24). The intervention components varied widely and included physician advice, counseling, educational booklets, health reminders, and video programs. One intervention focused on husbands’ smoking behaviors and wives’ avoidance behaviors (20). Two interventions focused on changes to husbands’ smoking behavior (21,22), and one focused on modifying behaviors through increasing self-efficacy among pregnant women (24). One intervention focused only on nonsmoking pregnant women’s avoidance behaviors with a pretest–posttest design (23).

### Prevalence and characteristics of pregnant women exposed to secondhand smoke

The prevalence of SHS exposure among pregnant women varied overall from 38.9% to 75.1%, with 17.0% to 73.0% exposed at home, 11.6% to 65.0% in the workplace, and 11.0% to 83.3% in public places (Table 1). In general, the prevalence estimates from population-based studies were high (60%–70%), except for Shanghai (a more developed city) and Macao (a city with a long history of tobacco control efforts). The prevalence reported by hospital-based studies generally matched the prevalence reported by population-based studies but was more varied.

The prevalence of SHS exposure varied by demographic variables such as age, education, income, and residence. Yang and coworkers (14) found that rural pregnant women (n = 784) in Sichuan province, China, were more likely to be exposed to SHS than were women in China’s urban environments (n = 397). Lee et al (19) found that younger pregnant women were more likely to report SHS exposure than were older pregnant women (48.2% [n = 485] vs 25.8% [n = 97]), and pregnant women with a university education were less likely to report SHS exposure than those without university education (*P* < .01).

The prevalence of exposure to SHS also varied across regions in China. Lee et al (19) reported that during 2000–2001 the daily SHS exposure was higher in a far north city than in the capital, Beijing (34.7% [n = 485] vs 12.3% [n = 166]). Fu et al (12) found that in Shanghai, the largest city in China and located in eastern China, new mothers reported occasional (8.9% of mothers) and everyday (32.9% of mothers) SHS exposure during pregnancy. Yang et al (14) reported that among 1,181 rural nonsmoking pregnant women in Chengdu, the capital city of Sichuan Province, in southwestern China, 75.1 % reported being exposed to SHS for at least 15 minutes daily, and 35.6% reported at least 1 hour of exposure daily in the previous week of. In summary, pregnant women in western areas were more likely to be exposed to SHS than their counterparts in northern areas.

### SHS exposure avoidance behaviors

Four descriptive studies reported avoidance behaviors of pregnant women exposed to SHS (12,15–17). Reports of avoidance behaviors at home ranged from 10.0% to 70.2%. The proportion of mothers who reported staying away from smokers at home ranged from 18.3% to 70.2%, and the proportion requesting their husbands to refrain from smoking in their presence ranged from 31.4% to 69.4%. The proportion of respondents who reported efforts to stop family members from smoking in their presence ranged from 16.7% to 47.8%, with 10.0% to 43.0% reporting that they opened windows at home. In the study by Loke et al (16), 74.5% of pregnant women moved away from smokers in public places; in another study, only 18.8% of pregnant women reported considering switching to another restaurant when they were in a restaurant where people were smoking (15). Among pregnant women whose husbands were current smokers in the study by Loke et al, 39% reported they often stepped away and 25% reported they sometimes moved away (16), whereas in the study by Luo et al, only 18.3% of pregnant women walked away when their husbands smoked in their presence (15). Luo et al conducted a survey in the Heishahuan district in Macao, a special administrative region in South China. They reported that 69.4% of pregnant women advised their husbands and 47.8% advised other family members to stop smoking in their presence. About half of pregnant women reported that they avoided SHS from their coworkers (50.9%) by removing themselves when people were smoking. The study by Lee (23) found that most pregnant women felt powerless and lacked self-efficacy to stop smokers from smoking in their presence.

### SHS interventions among pregnant women

Three studies in this review used an RCT (20–22) design and 2 used pre–post interventions (23,24). Loke and Lam (21) conducted an RCT in Guangzhou. The interventions included standardized advice provided by obstetricians, educational booklets, and health reminders to nonsmoking pregnant women. The authors found that husbands in the intervention group were more likely to report attempting to quit smoking and to reduce their level of smoking than husbands in the control group (30.0% vs 22.2%, *P* < .01 and 39.7% vs 17.7%, *P* < .01), respectively). Yang and Mao (22) also reported greater reductions in the number of cigarettes per day that husbands smoked in the intervention group compared with the control group (*P* < .01). Gao and colleagues (20) reported that a higher proportion of pregnant women walked away from smokers in the intervention group (44.5%) compared with pregnant women in the control group (39.7%) in a study conducted in 1996 in Guangzhou. In the study by Yang and Mao (22), hair nicotine concentration, a biomarker of long-term smoke exposure, was measured among pregnant women and was significantly lower in the intervention group than in the control group (*P* < .01). Lee found that an intervention designed to provide assertiveness training for pregnant women (eg, asking smokers to quit or to stop smoking in their presence) resulted in an increase in assertiveness after the intervention (23). In a pre–post intervention, Hu et al (24) showed that SHS exposure declined from 62.2% at preintervention to 33.8% at postintervention (*P* < .01). Hu et al also found that the self-efficacy of stopping all smokers other than husbands (eg, father-in-law, parents, relatives, visitors at home, coworkers) increased significantly (*P* < .01) but did not increase for stopping husbands from smoking (*P* > .05).

## Discussion

Results from this review show that despite a decade of tobacco control efforts that have resulted in reducing the prevalence of SHS exposure among pregnant women, the prevalence of exposure is still not at a safe or healthy level. In China, pregnant women’s exposure to SHS varies across the country, with some locations having a high level of exposure. Population-based studies conducted in Henan Province and Sichuan Province reported 60% to 70% of SHS exposure overall and 50% to 60% of SHS exposure among pregnant women at home. Some variation in prevalence across studies could reflect sample size, sample characteristics, data quality, data-gathering strategies, content of the survey or interview questions, or true differences in exposure. The variation could also reflect the regional differences in smoking prevalence among men. Previous studies indicated that smoking prevalence among men in China differs according to age group, living place, education level, occupation, and implementation of policies that ban smoking (25,26). The variation may also be caused by reporting bias among women because, in some areas, women may be hesitant to disclose their husband’s smoking behaviors. Macao, one of 2 special administrative regions (the other is Hong Kong) in China, had a low prevalence of SHS exposure among pregnant women, attributable in part to stronger tobacco control policies in Macao than in other developed countries. The lower prevalence in Macao is encouraging and demonstrates that the experiences of more advanced tobacco control policies can be applied to people with similar cultural backgrounds.

Despite efforts to disseminate information about the health effects of SHS exposure, studies suggest that women do not have or do not use methods for getting people with whom they have close contact to stop smoking in their presence. Although information on the adverse health effects of exposure to SHS during pregnancy has been widely disseminated in China (25), efforts by pregnant women to avoid SHS exposure, particularly at work and in public places, appear to be ineffective or suboptimal. However, nearly all pregnant women believe that smoking is harmful to the growth of the fetus (21). According to Lee (23), most pregnant women in China felt powerless and lacked self-efficacy to stop smokers from smoking in their presence. Women believed that they could not do anything to ensure a smoke-free environment at home and that they have even less impact in the workplace. Although these expectant mothers understood that exposure to SHS is harmful, they were anxious about disrupting family harmony if they asked their husbands or other family members to stop smoking (23). Therefore, future intervention focusing on improving the self-efficacy of pregnant women may enable them to stop smokers from smoking in their presence and may help to prevent SHS exposure in homes and serve as a breakthrough point for changing social norms in China.

To decrease the exposure of pregnant women to SHS as one breakthrough point of tobacco control, it is necessary to strengthen surveillance to be able to determine which areas may best benefit from intervention and prevention efforts. Currently, surveillance is scattered and unsystematic. We recommend that all regions use consistent questions and data collection methods for any future surveillance on exposure to SHS, so that comparisons between regions can be made. We also recommend that some attempt be made to validate the occurrence and extent of exposure.

Although China ratified the FCTC in 2005, the implementation of systematic tobacco control policy is lower than what FCTC requested (25), perhaps as a result of limited implementation of policies on smoke-free work and public areas. Numerous interventions to encourage smoking cessation among men have been conducted in China, but findings are inconclusive (27). For example, the study by Sun et al (28) of a smoking cessation intervention conducted in Beijing concluded that family support was a predictor of smoking cessation only because marital status was associated with abstinence; none of the components in the intervention, however, actually addressed the components of family support. Cultural factors may make cessation programs for men particularly challenging. For example, giving and sharing cigarettes is considered to be good for relationships, and smoking is a symbol of men’s freedom and independence in China (29). These cultural factors may preclude action on the part of women, who are expected to endure smoking among men without resistance. Nonsmoking pregnant women desire harmony and may perceive their husbands’ smoking as necessary for relieving work-related anxiety (23).

Although we found few intervention studies of exposure to SHS among pregnant women, we believe theory-driven interventions hold the greatest promise for intervention studies. The study by Loke and Lam (21) applied the Reasoned Action Model to the intervention, which provided advice to nonsmoking pregnant women on ways to help their smoking husbands quit. This study demonstrated the effect of the Reasoned Action Model intervention on quitting smoking. Because ethical concerns added difficulty to control arms, some interventions applied a pretest–posttest approach. Lee (23) applied the Health Belief Model in a pretest–posttest intervention, which resulted in more pregnant women asking smoking husbands and other smoking family members to stop smoking in their presence.

This literature review assists us in planning future studies examining SHS exposure among pregnant women in China. First, one major finding is the marked differences in the methods for collecting information and in the findings. We propose to standardize methods in surveillance by using standard questions for data collection and data-gathering strategies. For example, we will examine the utility and validity of collecting information through various means: quantitative interviews, self-administered questionnaires, qualitative interviews, focus groups, and biomarkers. Second, we will further study how to best apply the theories that have been proven effective in China, such as the Health Belief Model and Reasoned Action Model. Some studies that demonstrated effective interventions (20,22) in this review were not theory based. Our future examination of the findings from these studies might provide some basis for the development of theory-based interventions. Third, the use of interventions among pregnant women exposed to SHS is viewed as a breakthrough point to achieve the goal of a smoke-free home, a smoke-free office, and a change in the social norm of smoking.

Strengths of this study include that it is the first systematic review examining SHS exposure and interventions among pregnant women in China. This review summarized information available to date and revealed some areas in need of further investigation. The review is useful for providing suggestions about the content of future intervention studies; the findings may be relevant for other Southeast and Southwest Asian countries such as Vietnam (30) and Bangladesh. The situation in Vietnam is similar to that in China: smoking among men is socially acceptable, and there is considerable SHS exposure among pregnant women. In Bangladesh, 46.7% of reproductive-aged women reported exposure to SHS at home (31).

Our study has several limitations. First, all studies except one were based on self-report to assess levels of SHS exposure (23). There were no attempts to validate the occurrence or extent of exposure. Second, none of the intervention studies evaluated the implementation of policies to prevent SHS exposure at home or in offices. Most interventions focused on increasing women’s avoidance behaviors, such as opening windows and walking away from SHS. However, although the studies demonstrated reductions of SHS exposure among pregnant women, the optimal effective strategy for protection against SHS is to implement 100% smoke-free indoor areas (32). Avoidance by walking away, improving ventilation, and opening a window do not work to completely eliminate exposure. Future intervention studies need to design, implement, and evaluate the recommended approaches for protection. The use of focus groups and interviews will help to provide the content and methods for these approaches in a society where smoking is the norm.

Effective interventions for reducing SHS exposure among pregnant women are needed to help ensure the health of women and infants. The content of these interventions needs to be culturally relevant and should take into account input from both pregnant women and their husbands who smoke. The use of theory-driven and community-based interventions could provide the foundation for these programs and strive to change the culture of smoking around pregnant women.
